# Establishment of a TaqMan-based quantitative real-time PCR for the detection of porcine parvovirus

**DOI:** 10.3389/fvets.2026.1736303

**Published:** 2026-03-06

**Authors:** Zhiqiang Hu, Maosi Xu, Guoqiang Tang, Ran Guan, Xingsheng Lai, Kelei Zhou, Hao Li, Yadong Jin, Jingang Zhao, Wei Xu, Zengwen Huang

**Affiliations:** 1 College of Animal Science, Xichang University, Xichang, China; 2 Key Laboratory of Animal Epidemic Disease Detection and Prevention in Panxi District, Xichang University, Xichang, China; 3 Shandong Zhongxin Food Group Co., Ltd., Jinan, China; 4 Animal Disease Prevention and Control Center, Bureau of Agriculture and Rural Affairs of Liangshan Yi Autonomous Prefecture, Liangshan, China; 5 Animal Disease Prevention and Control Center, Bureau of Agriculture and Rural Affairs of Xichang City, Xichang, China; 6 MOA Key Laboratory of Animal Virology, Center for Veterinary Sciences, Department of Veterinary Medicine, College of Animal Sciences, Zhejiang University, Hangzhou, China

**Keywords:** clinical samples, NS1 gene, porcine parvovirus, sensitivity, TaqMan-qPCR

## Abstract

Porcine Parvovirus (PPV) is a non-enveloped DNA virus that predominantly induces reproductive disorders in swine. The ongoing emergence of novel PPV variants and the frequent co-infections with other viruses have led to significant economic losses within the swine industry. This study, utilizing 31 previously reported complete PPV genome sequences, identified a conserved fragment of the PPV-NS1 gene through homology analysis. A TaqMan-based real-time quantitative PCR (TaqMan-qPCR) detection method was developed targeting this specific fragment. Sensitivity assessments determined a detection limit of 8.5 copies/μL for standard plasmids. Specificity assessments showed no cross-reactivity with 10 other prevalent swine pathogens. The coefficients of variation for both intra-assay and inter-assay repeatability tests were both under 1%, demonstrating high reproducibility. Moreover, an analysis involving 32 clinical samples was conducted to compare the detection outcomes of the developed method with those obtained from a commercial kit. The findings demonstrated that the established method achieved a relative sensitivity of 100% and a relative compliance rate of 75%, suggesting its potential as an alternative to the commercial kit. In summary, the TaqMan-qPCR method developed in this study exhibits high sensitivity and specificity, making it ideal for detecting various clinical samples. It also provides a valuable tool for monitoring PPV and examining its epidemiological traits.

## Introduction

Porcine Parvovirus (PPV) is a non-enveloped virus classified within the *Parvoviridae* family, recognized as a principal pathogen responsible for reproductive disorders in swine (1). The viral genome consists of approximately 5.0 kb of single-stranded DNA and includes two main open reading frames, ORF1 and ORF2. These ORFs encode non-structural proteins (NS1, NS2, NS3) and structural proteins (VP1, VP2, VP3) respectively, with VP2 being the primary immunogenic protein (2). PPV is globally prevalent and significantly contributes to reproductive failures in swine. Research indicates that PPV infection is not restricted by the age, sex, or breed of pigs; any pig can be infected (3, 4). However, primiparous sows exhibit a higher incidence of reproductive disorders following infection (3, 4). In sows, symptoms of PPV infection encompass abortion, stillbirths, and fetal mummification, which can significantly contribute to reproductive disorders across entire pig herds (5). In piglets, PPV infection may lead to stunted growth, compromised immunity, and heightened susceptibility to other diseases, thereby adversely affecting the economic viability of pig farming (6). With the increasing intensification of pig farming in China, the prevalence of PPV has garnered heightened attention. Co-infections of PPV with Porcine Circovirus Type 2 (PCV2) and Porcine Reproductive and Respiratory Syndrome Virus (PRRSV) are well-documented, indicating PPV’s potential significance in the Porcine Respiratory Disease Complex (PRDC) (7–9). PPV is prevalent not only in domestic pigs but also in wild boars, which can act as virus reservoirs and threaten domestic pig populations. Research conducted in Germany and Turkey has identified wild boars as carriers of various porcine viruses, including PPV, underscoring the critical importance of continuous wildlife monitoring to mitigate the risk of viral transmission between domestic swine and wild fauna (10, 11). Consequently, the rapid and precise detection of PPV presents a significant challenge for the swine industry.

According to the Agricultural Industry Recommended Standard of “Diagnostic techniques for porcine parvovirus infection” (NY/T 4137–2022) issued in China in 2022, the conventional PCR technology targeting the PPV-NS1 gene is currently one of the gold standard methods for PPV diagnosis. Molitor et al. developed a conventional PCR method targeting the PPV-VP2 gene, achieving a detection threshold of 100 fg/reaction (12). Cui et al. have optimized traditional PCR techniques. Established a conventional PCR method based on the PPV-NS1 gene in 2014, with a sensitivity of 5.6 × 10^3^ copies/μL (13). Yue et al. developed a multiplex PCR technique grounded in conventional PCR principles, enabling the concurrent rapid detection of PCV2, PPV, PRV, and PRRSV in clinical samples, achieving a minimum detection threshold of 68–680 copies/reaction for the PPV genome (14). However, as an established molecular detection method, conventional PCR lacks quantitative capacity and generally requires post-amplification electrophoretic analysis, which prolongs the detection cycle and carries a potential risk of cross-contamination. With the increasing intensification of the pig industry in China, most large-scale pig farms have established on-site diagnostic laboratories for pathogen and antibody detection. Therefore, qPCR technology, characterized by prominent advantages including accurate quantitative capability, rapid detection speed, and reduced contamination risk via a closed-tube system, has been increasingly applied in current clinical veterinary diagnostics. This study aims to develop and refine probes and primers targeting conserved genomic regions of various PPV strains to establish a TaqMan-qPCR method for detecting clinically suspected PPV infections. This approach not only facilitates swift and accurate diagnosis but also provides robust technical support for early detection and epidemiological investigations of PPV.

## Materials and methods

### Primers and probes

**Figure 1:**
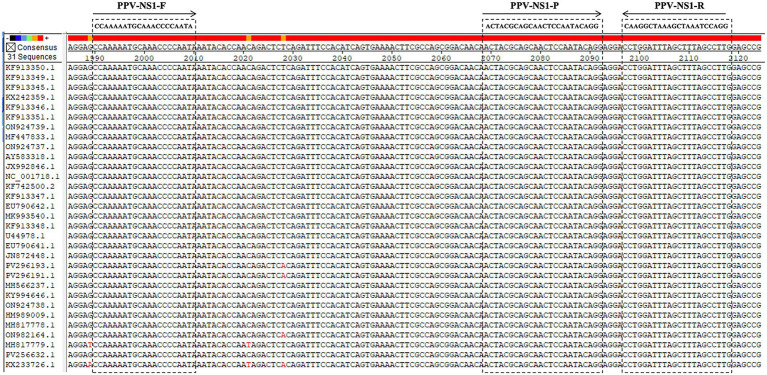
Homology analysis of 31 PPV strains.

Utilizing the complete genome sequences of 31 PPV strains sourced from GenBank, we conducted a homology analysis employing DNAStar software, as depicted in Figure 1. Primers and a TaqMan probe were designed with Primer Express 3.0 to target conserved sequence regions. The sequences of the designed primers are as follows: 5′-CCAAAAATGCAAACCCCAATA-3′ (forward), and 5′-CAAGGCTAAAGCTAAATCCAGG-3′ (reverse), while the TaqMan probe is 5′-FAM-ACTACGCAGCAACTCCAATACAGG-MGB-3′. The amplified gene fragment was 129 base pairs in length. Both the primers and the TaqMan probe were synthesized and labeled by Sangon Biotech (Shanghai) Co., Ltd.

### Standard plasmid

The PPV-NS1 gene sequence was amplified, synthesized, and cloned into the pUC57 vector to create the pUC57-PPV standard plasmid. Positive plasmids were quantified via UV–Vis spectrophotometry, and their copy number was determined using a specific formula (15). A 10-fold serial dilution was performed, producing concentrations from 8.5 × 10^9^ to 8.5 × 10^0^ copies/μL, which were subsequently stored at −20 °C for future use.


$$\text{Plasmid copies}/\mathrm{\mu L}=\frac{\left(6.02\times 10^{23})\times (\mathrm{Xng}/\mathrm{\mu L}\times 10^{-9}\right)}{\text{plasmid length}\;(\mathrm{bp})\times 660}$$

### Reaction parameters of the TaqMan-qPCR method

All qPCR reaction systems were prepared in a total volume of 20 μL, following the instructions of the commercial kit (Q513, Vazyme, Nanjing, China). The 20 μL reaction mixture included 10 μL of 2× AceQ Universal U + Probe Master Mix V2, 0.4 μL each of forward and reverse primers (10 μmol/L), 0.2 μL of probe (10 μmol/L), 2 μL of template, and deionized water to achieve the final volume. The reaction conditions comprised an initial 2-min incubation, followed by a 5-min denaturation at 95 °C, and 40 cycles of 95 °C for 10 s and 60 °C for 30 s.

### Assessing sensitivity and establishing standard curves

Standard plasmids, diluted serially by tenfold from 8.5 × 10^9^ to 8.5 × 10^0^ copies/μL, were used as amplification templates. Using the amplification kinetics curve obtained, we plotted the logarithm of the copy number of the positive standard’s copy number on the *x*-axis and the cycle threshold (Ct value) on the *y*-axis. This enabled the development of the TaqMan-qPCR standard curve and the derivation of its linear regression equation.

### Evaluating specificity

Specificity was assessed using the established TaqMan-qPCR method to detect nucleic acids in positive samples of various viruses, including African Swine Fever Virus (ASFV), PRRSV, Porcine Epidemic Diarrhea Virus (PEDV), PCV2, PCV3, Classical Swine Fever Virus (CSFV), Transmissible Gastroenteritis Virus of Swine (TGEV), Porcine Rotavirus (PoRV), Pseudorabies Virus (PRV), Japanese Encephalitis Virus (JEV), and PPV. Sterilized deionized water served as the negative control.

### Evaluating reproducibility

Reproducibility was assessed by amplifying templates with pUC57-PPV standard plasmids at concentrations ranging from 8.5 × 10^5^ to 8.5 × 10^1^ copies/μL. Three batches of tests were conducted, each containing three replicates per dilution. Statistical analysis of Ct values was conducted to determine intra-group and inter-group coefficients of variation, evaluating the method’s repeatability and stability.

### Comparative analysis of the established TaqMan-qPCR assay versus a commercial kit using clinical samples

A total of 32 clinical tissue samples were collected from aborted fetuses in a pig farm, and then send to our laboratory for PPV diagnosis, including mesenteric lymph nodes (*n* = 8), lungs (*n* = 12) and kidneys (*n* = 12). The samples were evaluated using both the TaqMan-qPCR method and a commercial qPCR kit (GM11016, Guanmu Biotechnology, Changsha, China). The efficacy of the developed TaqMan-qPCR method was evaluated in comparison to the commercial PCR method by determining relative sensitivity as [(true positive)/(true positive + false negative)] × 100% and relative specificity as [(true negative)/(true negative + false positive)] × 100%.

To further validate the accuracy of the method established in this study, 12 samples were selected from those previously analyzed using the established TaqMan-qPCR method. These samples included negative samples (*n* = 3), samples with Ct < 30 (*n* = 3), samples with 35 > Ct ≥ 30 (*n* = 3), and samples with Ct ≥ 35 (*n* = 3). These were subsequently tested using the conventional PCR method as recommended by the Agricultural Industry Recommended Standard “Diagnostic Techniques for Porcine Parvovirus Infection” (NY/T 4137–2022).

### Clinical sample testing

A total of 215 clinical samples were collected by farmers from a breeding pig farm in Sichuan Province and then submitted to our laboratory for further detection and analysis. These specimens included 54 oropharyngeal swabs, 72 blood samples and 89 sow aborted materials, of which the latter consisted of aborted fetal tissues, placental membranes and amniotic fluid from sows. All samples (300 μL per sample) were subjected to nucleic acid extraction using the NPA-96E automatic nucleic acid extractor manufactured by Hangzhou Boer Technology Co., Ltd. Then, 2 μL of the purified DNA was subjected to the developed qPCR assay. The pUC57-PPV standard plasmid was employed as the positive control, and double-distilled water (ddH_2_O) was used as the negative control. A result was defined as positive when the Ct value was lower than 40. The positive rates of PPV in different sample types were presented as absolute and relative frequencies (%), with 95% confidence intervals (CIs) calculated via the normal approximation method.

## Results

### Standard curve, sensitivity and specificity

**Figure 2:**
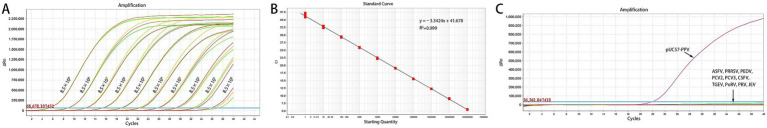
**(A, B)** Amplification curve and standard curve of the TaqMan-qPCR method for detecting PPV. **(C)** Amplification curves of specificity testing among porcine viruses.

The TaqMan-qPCR method demonstrated a strong linear correlation in detecting plasmid standards across concentrations from 8.5 × 10^9^ to 8.5 × 10^0^ copies/μL, with the minimum detectable concentration being 8.5 × 10^0^ copies/μL (Figure 2A). The standard curve exhibited a slope of −3.424 with a correlation coefficient (*R*^2^) of 0.999, an amplification efficiency of 95.92%, and was described by the equation *Y* = −3.424*x* + 41.678 (Figure 2B).

This validated TaqMan-qPCR method was subsequently employed to detect cDNA from various porcine pathogens. Figure 2C demonstrated that amplification occurred solely for the pUC57-PPV standard plasmid, with no amplification signals for other common viruses such as ASFV, PRRSV, PEDV, PCV2, PCV3, CSFV, TGEV, PoRV, PRV, and JEV. The results indicated that the TaqMan-qPCR detection method is highly specific and does not cross-react with common porcine pathogens.

### Reproducibility testing

Table 1 and showed that the intra-group coefficient of variation ranged from 0.20 to 0.83%, and the inter-group coefficient of variation ranged from 0.17 to 0.82%, demonstrating the method’s excellent reproducibility.

**Table 1:** Intra-assay and inter-assay reproducibility test of the TaqMan-qPCR.

Template concentration (copies/μl)	Intra-assay variation						Inter-assay variation					
	Ct values			Average value	Standard deviation	CV	Ct values			Average value	Standard deviation	CV
8.50 × 10^5^	18.35	18.25	18.25	18.28	0.05	0.26%	18.23	18.16	18.15	18.18	0.04	0.20%
8.50 × 10^4^	22.58	22.47	22.52	22.52	0.04	0.20%	22.73	22.75	22.82	22.77	0.04	0.17%
8.50 × 10^3^	25.36	25.23	25.40	25.33	0.07	0.29%	25.45	25.21	25.32	25.33	0.10	0.39%
8.50 × 10^2^	28.37	28.92	28.55	28.61	0.23	0.80%	28.25	28.57	28.82	28.55	0.23	0.82%
8.50 × 10^1^	32.42	31.98	31.78	32.06	0.27	0.83%	32.15	31.78	32.31	32.08	0.22	0.69%

### Comparative analysis of the established TaqMan-qPCR assay versus a commercial kit using clinical samples

Table 2 demonstrated that the TaqMan-qPCR analysis of clinical samples achieved a relative sensitivity of 100%, matching the sensitivity of the commercial kit. The TaqMan-qPCR method demonstrated a 75.00% compliance rate, indicating its potential as an alternative to the commercial kit. The results of the comparative study with the conventional PCR method were presented in Supplementary Table S1. Notably, 9 samples yielded results consistent with the established TaqMan-qPCR method, including those with Ct < 35 and negative samples. In contrast, all samples with Ct ≥ 35 tested negative using the conventional PCR. These findings suggest that the accuracy of the established method satisfies clinical requirements; however, the sensitivity of the conventional PCR method is inferior to that of the established qPCR method.

**Table 2:** Comparison between the TaqMan-qPCR method and the commercial qPCR kit with clinical samples.

TaqMan-qPCR	Commercial qPCR kit		Total
	+	−	
+	18	8	26
−	0	6	6
Total	18	14	32

Relative sensitivity = 18/18 = 100%.

Relative specificity = 6/14 = 42.86%.

Compliance rate = 24/32 = 75.00%.

### Clinical sample testing

As shown in Table 3, the PPV positive rate in oropharyngeal swabs was 16.67% (9/54, 95% CI: 6.83–29.90%), followed by 9.72% (7/72, 95% CI: 2.75–18.39%) in blood samples. The highest PPV positive rate was detected in sow aborted materials, reaching 25.84% (23/89, 95% CI: 16.56–36.96%), indicating that clinically, sow aborted materials serve as the primary sample type for PPV diagnosis, while blood samples and oropharyngeal swabs are used as auxiliary specimens for screening and supplementary diagnosis.

**Table 3:** Positive rates of PPV in different clinical samples.

Sample type	No. of positive sample	No. of total sample	Positive rate	95% CI
Oropharyngeal swabs	9	54	16.67%	7.94–25.40%
Blood	7	72	9.72%	3.26–16.18%
Aborted materials of sows	23	89	25.84%	16.86–34.82%

## Discussion

The qPCR technology is highly suitable for rapid clinical diagnostics in intensive pig farms, owing to its operational flexibility, reduced time requirements, and high sensitivity. Currently, numerous multiplex methods employing qPCR have been reported, which include PPV detection. Bhattacharjee et al. introduced a dual qPCR method utilizing SYBR Green, achieving a detection threshold of 10 copies/μL for the PPV-VP2 gene (16). The SYBR Green-based qPCR method has reduced specificity and an increased likelihood of false-positive results (17). Consequently, the TaqMan-based qPCR method is predominantly employed in the detection of swine diseases. For instance, Quan et al. developed a quadruple qPCR assay with TaqMan probes to detect PCV1, PCV2, PRV, and PPV, utilizing the PPV-VP2 gene for PPV detection with a sensitivity of 10 copies/μL (18). Chen et al. developed a multiplex qPCR assay for the concurrent detection of four viruses implicated in porcine reproductive disorders, namely PCV2, PCV3, PPV, and PRV. The assay targets the NS1 gene for PPV detection, maintaining a consistent detection threshold (19). Shin et al. developed a duplex real-time PCR assay for the detection of Aujeszky’s disease virus (ADV) and PPV, utilizing the NS1 gene as the detection target for PPV. The limit of detection (LOD) was determined to be 1 TCID_50_/mL, demonstrating a sensitivity 10 times greater than that of conventional PCR (20). While multiplex PCR facilitates the simultaneous identification of multiple viral pathogens in a single reaction, thereby conserving both sample material and time, the primer design process remains intricate and prone to cross-interference. To further improve the sensitivity of PPV detection methods while ensuring accuracy and stability in clinical diagnostics, a singleplex assay based on the qPCR principle is a more advantageous approach. Several studies have reported on single qPCR methods for PPV detection. For instance, Wilhelm et al. developed a SYBR Green qPCR assay targeting the PPV-VP2 gene, achieving a detection limit of 60 copies/μL (21). Similarly, Song et al. employed primers and probes specific to the NS1 gene in a TaqMan-qPCR approach, achieving a detection threshold of 100 copies/μL for PPV (22). Gava et al. developed a TaqMan-qPCR assay targeting the ORF3 gene of PPV4, achieving a detection limit of 95 copies/μL, which represents a tenfold improvement over the conventional PCR method (23). Similarly, Li et al. and Sun et al. developed SYBR Green qPCR methods targeting the capsid gene of PPV7 and PPV6, achieving detection limits of 35.6 copies/μL and 47.8 copies/μL respectively, with a sensitivity 1,000 times greater than that of traditional PCR (24, 25). In this study, we utilized the TaqMan method targeting the NS1 gene of PPV to develop a detection method with a sensitivity threshold of 8.5 copies/μL. This threshold is lower than those reported for previous singleplex or multiplex PCR assays, thereby meeting the sensitivity requirements for clinical detection. Additionally, the intra-assay and inter-assay reproducibility of the singleplex qPCR assay established in this study were both within 1%, which is lower than that of the reported multiplex PCR methods and aligns with the stability requirements for clinical diagnostics (16, 18, 19). Furthermore, compared with multiplex PCR methods, the singleplex PCR assay offers simpler operation and troubleshooting, rendering it more suitable for clinical demands for process controllability. To assess the reliability of the newly established method for clinical sample detection, a comparative analysis was conducted against conventional PCR and commercial diagnostic kits. The findings indicated that conventional PCR was unable to detect PPV in samples with a Ct value exceeding 35, likely due to its higher LOD relative to qPCR. This observation aligns with existing literature, which suggests that the LOD of conventional PCR is typically 10–1,000 times greater than that of qPCR (20, 22–25), a conclusion corroborated by our study’s results. Consequently, in clinical settings, samples exhibiting Ct values between 35 and 40 are often classified as suspect, necessitating further observation or the employment of other detection methods for verification and confirmation. Furthermore, the reliability of the established method was substantiated through the analysis of a substantial number of clinical samples, consisting of different sample types.

Furthermore, existing PCR detection techniques for PPV predominantly target the NS1 and VP2 genes. The NS1 gene, which encodes a protein essential for viral replication, is highly conserved due to slow evolutionary rate and purifying selection (26, 27). In contrast, the VP2 gene, encoding the main immunogenic capsid protein exposed to host immune pressure, is more susceptible to mutations and genetic variation (26, 27). In this study, we analyzed 31 reported full-length gene reference sequences of PPV and compared them with primer sequences, observing the conserved region of NS1 genes with 100% homology. This finding further substantiates the efficacy of this method in the clinical detection of PPV.

In conclusion, this study established a highly sensitive and specific TaqMan-qPCR method for PPV detection. This method is applicable to the detection of various clinical samples and can provide an effective tool for the monitoring of PPV and the research on its epidemiological characteristics.

## Data Availability

The original contributions presented in the study are included in the article/Supplementary material, further inquiries can be directed to the corresponding author.
